# Attachment status is associated with grey matter recovery in adolescent anorexia nervosa: Findings from a longitudinal study

**DOI:** 10.1111/ejn.15614

**Published:** 2022-02-12

**Authors:** Lukas Lenhart, Manuela Gander, Ruth Steiger, Angieszka Dabkowska‐Mika, Stephanie Mangesius, Nina Haid‐Stecher, Martin Fuchs, Anna Buchheim, Kathrin Sevecke, Elke Ruth Gizewski

**Affiliations:** ^1^ Department of Radiology Medical University of Innsbruck Innsbruck Austria; ^2^ Department of Neuroradiology Medical University of Innsbruck Innsbruck Austria; ^3^ Neuroimaging Research Core Facility Medical University of Innsbruck Innsbruck Austria; ^4^ Department of Child and Adolescent Psychiatry Medical University of Innsbruck Innsbruck Austria; ^5^ Department of Child and Adolescent Psychiatry Tirol Kliniken Hall in Tirol Austria; ^6^ Institute of Psychology University of Innsbruck Innsbruck Austria

**Keywords:** adolescence, anorexia nervosa, attachment, childhood trauma, grey matter

## Abstract

The aim of the present study was to investigate whether grey matter (GM) reductions in acute anorexia nervosa (AN) are (i) valid for adolescents (age 14–18 years), (ii) reversible following short‐term psychotherapeutic and nutritional therapy and (iii) depend on psychological components like attachment trauma. 3T MRI including a high‐resolution T1 MPRAGE was performed in 22 female adolescents in the acute state of AN (age: 15.2 ± 1.2 years) and after weight restoration (duration: 2.6 ± 1 months, *n* = 18) and compared with 18 gender‐matched healthy controls. The Adult Attachment Projective Picture System was used to classify resolved and unresolved attachment patterns. GM decreases were localized in extensive cortical areas including the insula, prefrontal and cingulate cortices as well as subcortical regions during acute AN, which partially increased after therapy with a relative sparing of the hippocampus and parahippocampal gyrus. The resolved group showed more GM recovery in regions of the left hippocampus and parahippocampal gyrus, bilateral cerebellar regions, right precuneus and adjacent cingulate cortices relative to the unresolved pattern. Structural anomalies in adolescent AN that recovered after treatment may be primarily the consequence of malnutrition, whereas several regions did not display significant recovery. Attachment status seems to influence region‐specific GM recovery.

## Graphical abstract

Initial grey matter reductions in regions that are considered as crucial components of anorexia nervosa are shown to mainly recover after weight restoration in adolescence. Nevertheless, several brain regions remain at decreased levels after treatment. Patients with attachment trauma demonstrate less grey matter recovery in regions of the right precuneus/posterior cingulate, as well as the left hippocampus, left parahippocampal gyrus and cerebellar areas after weight recovery suggesting that attachment patterns might contribute to region‐specific grey matter recovery.

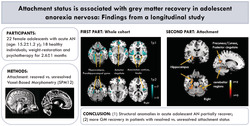

**Abbreviations**
AN: anorexia nervosa
BMI: body mass index
EDI‐2: Eating Disorder Inventory‐2
GM: grey matter
HC: healthy control participants
MNI: Montreal Neurological Institute
MRI: magnetic resonance imaging
SCID‐I: Structured Clinical Interview for DSM‐IV
Tp1: timepoint 1
Tp2: timepoint 2

## 1 INTRODUCTION

Anorexia nervosa (AN) is a serious psychiatric disorder reaching a lifetime prevalence up to .9% in females (Hudson et al., 2007). This condition commonly develops during adolescence, which is a crucial time for personal, social, and biological development (Smink et al., 2012). Patients with AN often report traumatic experiences during their upbringing like emotional abuse, parental divorce or mental health problems of their parents (Maxwell et al., 2017). The quality of an attachment relationship to their caregivers significantly affects the way adolescents deal with the exposure to adverse life events (Solomon & George, 2011).

Based on the Adult Attachment Projective Picture System (AAP) manual four attachment representations can be distinguished (George & West, 2012). A *secure attachment representation* is related to greater resilience under conditions of extreme stress or trauma. Secure individuals have experienced their caregivers as available and sensitive towards their attachment needs, and thus, they are capable to reach out for comfort when confronted with attachment‐related distress. Consequently, they show more successful coping strategies and better skills in conflict management (Bizzi et al., 2015; George & West, 2012). Insecurely attached individuals, on the other hand, experienced ruptures in their attachment relationships. Individuals with an *insecure‐dismissing representation* experienced their caregiver's rejection in response to their attachment signals. They predominantly demonstrate deactivating strategies that help to maintain a distance in relationships. *Insecure‐preoccupied individuals* were confronted with inconsistent responses of their caregivers, and they primarily employ cognitive disconnection defensive strategies to confuse or obscure attachment relationships in order to prevent the breakthrough of traumatic attachment material. These three attachment patterns are considered resolved as individuals remain organized when confronted with stressful attachment situations (George & West, 2012).

In contrast to individuals with an organized/resolved attachment representation (secure, insecure‐dismissing, insecure‐preoccupied), *disorganized/unresolved* individuals fail to find solace and comfort in face of threatening or traumatic attachment situations, and thus, these experiences become too overwhelming and leave them in a state of dysregulation and helplessness (Laible, 2007). Individuals with an unresolved attachment status are either faced with threatened abandonment (i.e., parental abandonment, loss or suicide) or potential danger by their caregiver (i.e., physical or sexual abuse) (Buchheim & Diamond, 2018; Buchheim & George, 2011). Such disruptions in the attachment relationship are distressing and frightening, and they set the groundwork for developing attachment trauma (George & West, 2001). Whereas secure attachment is related to greater resilience, insecure and unresolved/disorganized attachment leads to helplessness and does not help the adolescent to restore safety and comfort (Laible, 2007).

In the clinical context, unresolved attachment is associated with a high risk for the development of psychological disorders and an increased symptom severity (Bizzi et al., 2015). Furthermore, attachment organization can influence the psychotherapy process and the outcome (Levy et al., 2018). One of the main goals of psychotherapy is to establish a secure base that allows patients to explore their self more fully and safely, their relationships and contents of their mind. Furthermore, psychotherapists can provide the patient with a temporary safe attachment figure that the patient can seek out for protection and comfort in times of distress (Levy, 2013; Levy et al., 2018). This might be of particular importance to patients with AN because they often lack confidence to cope with negative feelings (Burns et al., 2012) and they have difficulties to rely on attachment figures for safety and comfort (Ringer & Crittenden, 2007). Their preoccupation with weight and shape, their obsessive control of their eating behaviours and their resentment of food provides them with a false sense of control over their lives and replaces their needs for a secure base (Gander, Diamond, et al., 2018; Latzer & Hochdorf, 2005). For AN patients with an unresolved attachment status, it might be particularly difficult to respond to psychotherapeutic treatments that do not focus on interpersonal aspects. They often demonstrate underdeveloped capacities to regulate their negative emotions in relationships, and they show higher levels of emotional distress, impulsivity and social impairment (Laible, 2007). When facing stressful or traumatic situations, they are flooded by feelings of fear and threat because they are unable to find solace and comfort in attachment figures. For clinicians, AN patients with an unresolved attachment status can be very challenging because they tend to experience the clinician as a source of both danger and security, and thus, these individuals often dropout of therapy or show negative treatment outcomes (Gander, Sevecke, & Buchheim, 2018).

Over the last two decades, researchers have begun to examine neurobiological substrates that contribute to the course and outcome of eating disorders with a growing use of quantitative neuroimaging methods to examine the relationship between structural brain abnormalities and psychopathology (Kaye et al., 2009). Several studies have revealed brain alterations in AN patients and concluded that reduced grey matter (GM) and white matter volumes as well as ventricular enlargement are linked to malnourishment and completely recover after weight restoration in adults (Seitz et al., 2014, 2016). In addition to global brain volume reductions, an increasing number of analyses have applied voxel‐based morphometry in adult patients with acute AN to localize regional patterns of brain atrophy in a more precise way (King et al., 2015; Mühlau et al., 2007; Seitz et al., 2014; Titova et al., 2013). Most consistently, relative GM decreases were found in larger frontal and subcortical volumes as well as the cingulate gyrus and cerebellum (for review, see King et al., 2018). However, only a few studies were conducted in adolescents. Especially during adolescence, neural plasticity and circuitry are known to be under ongoing maturation and affected by social and environmental influences (e.g., interpersonal difficulties or socioeconomic status) resulting in potential medical complications and psychological impairments (Herpertz‐Dahlmann, 2015; Swanson et al., 2011). Furthermore, it has been hypothesized that the maturing brain of an adolescent is particularly vulnerable to damage caused by extreme restriction of food intake (Herpertz‐Dahlmann, 2015). Further, GM deficits appear to be more pronounced in adolescents than adults. Adolescents may differ in the persistence of these alterations as well as in the course and outcome of their disease (Seitz et al., 2016). Existing studies in adolescent AN cohorts suggested that GM volume reductions partially normalize during weight restoration therapy (Bernardoni et al., 2016; Bomba et al., 2015; Castro‐Fornieles et al., 2010; Friederich et al., 2012; King et al., 2015; Mainz et al., 2012). However, structural abnormalities were reported to persist in regions including the anterior cingulate, supplementary motor area and precuneus after nutritional therapy in adults (Friederich et al., 2012; Joos et al., 2011; Mühlau et al., 2007), as well as clusters in the anterior cingulate, caudate nuclei and right hippocampus following short‐term (i.e., 2–3 months) weight restoration in adolescent patients (Martin Monzon et al., 2017).

The ventral‐limbic system, which includes the amygdala, insula, ventral striatum, ventral anterior cingulate and orbitofrontal cortex, has been proposed to modulate the emotional significance of a stimulus and to generate a corresponding affective state or a behavioural response. Another important circuit is the dorsal cognitive system consisting of regions such as the hippocampus, dorsal anterior cingulate and prefrontal cortex, which are involved in the execution of functions such as attention control and cognitive flexibility as well as in the regulation of affective states (Phillips et al., 2003a, 2003b). Alterations, particularly in these regions, could explain symptoms of AN patients such as inhibition, anxiety, depression, body image distortion and alexithymia. Therefore, less GM recovery in these regions may lead to persistent emotional as well as cognitive disorders.

Despite a growing number of neuroimaging studies investigating structural alterations in acute AN (Seitz et al., 2016), there is still a relative lack of (i) longitudinal studies that hold the potential to differentiate between state‐related effects, for example, due to acute undernutrition, and more enduring abnormalities, (ii) studies in younger age groups (i.e., 14–18 years) and (iii) studies that included psychological components like attachment status, which was shown to impact therapeutic response of AN patients regarding weight restoration. In this context, research focusing on unresolved attachment status is still underrepresented in the literature. Therefore, we aimed to investigate in the first part of the study altered brain structures in the acute disease state and after inpatient psychotherapeutic treatment and weight restoration (body mass index [BMI] for‐age ≥ 5th percentile) in a cohort of underweight adolescents with acute AN (i.e., age range from 14 to 18 years). We assumed that patients with AN show reduced GM volumes particularly in brain areas that could be involved in the symptomatology of eating disorders such as the striatum, prefrontal and cingulate cortices and that these regions recover to near normal levels of healthy controls (HCs) after therapy depending on the individual therapeutic response. Evidence from the literature suggests that a patient's attachment status is related to therapeutic response. In particular, the unresolved attachment status is associated with worse treatment outcome (de Paoli et al., 2017; Gander, Sevecke, & Buchheim, 2018). Therefore, the second study part explored whether variances in GM recovery may be related to the patients' attachment status. As attachment‐related dysregulation under conditions of extreme stress is hypothesized to manifest in certain brain structures that are associated with emotion regulation deficits (Baldaçara et al., 2008; Benetti et al., 2010), we hypothesized that patients with an unresolved attachment status show less GM recovery after short‐term therapy compared with those with a resolved attachment status (secure, insecure‐dismissing, insecure‐preoccupied).

## 2 MATERIALS AND METHODS

### 2.1 Participants

The initial sample for this study consisted of 27 female inpatients from the Department of Child and Adolescent Psychiatry aged from 14 to 18 years and 23 adolescent controls who were recruited in different areas of Southern Germany and Austria. All patients were diagnosed with restrictive AN based on the Structured Clinical Interview for DSM‐IV (SCID‐I, Wittchen et al., 1997). Prior to the scanning procedure, we evaluated psychological and medical exclusion criteria. In controls, we assessed the SCID‐I interview to exclude adolescents with a present or a history of a mental disorder (eating disorder, psychosis, anxiety disorders, etc.). Both AN patients and HC underwent multiple laboratory analyses and a standard physical examination to ensure the fulfilment of the inclusion criteria. The presence of medical conditions or metabolic diseases (i.e., acute or chronic somatic or functional diseases like strokes, tumours, heart conditions, a history of head trauma or fainting) leads therefore to the exclusion of patients and controls. All patients meeting the inclusion criteria experienced amenorrhea. Extremely underweight AN patients requiring paediatric treatment for medical stability and improvement of cognitive functioning prior to psychiatric inpatient treatment were excluded from study participation. From a total of 27 AN patients, 5 were excluded due to loss of neuropsychological follow‐up (*n* = 3) and movement artefacts during the magnetic resonance imaging (MRI) scan (*n* = 2). Additionally, 5 controls with incidental findings on the MRI scan (multiple sclerosis and hydrocephalus; *n* = 2), a history of AN as assessed with the SCID‐I interview (*n* = 1), as well as conditions that would have reduced the image quality significantly such as artefacts due to movement (*n* = 1) and dental brace (*n* = 1) were excluded. Further exclusion criteria for all participants were an intelligence score < 85 assessed by the Hamburg Wechsler Intelligence Scale (Petermann & Petermann, 2008), an insufficient knowledge of the German language for the psychological tests and questionnaires, a history of substance abuse and contraindications for MRI. The final study sample consisted of 22 patients (mean age: 15.2 ± 1.2 years) and 18 gender matched controls (mean age: 16.8 ± .9 years).

Patients were recruited within the first week of their admission to the specialized unit for eating disorders at the Department of Child and Adolescent Psychiatry. Inpatient psychiatric treatment included psychoeducation, individual psychotherapeutic treatment and family‐based therapy. Medical supervision included a close monitoring of altered vital signs and abnormal laboratory findings like electrolyte or electrocardiogram changes. The refeeding plan for each patient varied from a total intake of 2400 to 3000 kcal/day depending on markers of medical instability. Patients' meal intakes and mental conditions were reviewed daily. During hospitalization, patients attended a hospital‐based school, besides regularly psychotherapeutic and adolescent group sessions. They did not have therapy that was attachment focused whilst they were in hospital.

After reaching medical stability, patients had the first MRI brain scan (timepoint 1 [Tp1], *n* = 22), and they completed the questionnaires and the clinical interviews. After weight restoration (BMI‐for‐age ≥ 5th percentile), patients were asked to participate in a second MRI scan (timepoint 2 [Tp2]; *n* = 18) on average 2.6 ± 1 months following the first session. HC underwent one MRI scan.

The study was carried out according to the Declaration of Helsinki and received ethical approval by the Hospital Ethics Committee (AN2015‐0036). Informed consent was received from all participants and their parents/legal guardians.

### 2.2 Measures

#### 2.2.1 Eating disorder diagnosis and symptomatology

The Structured Clinical Interview for DSM‐IV (SCID‐I, German translation: Wittchen et al., 1997) was used to diagnose AN. It was administered by trained clinical psychologists at our psychiatric unit. The SCID‐Interview is often considered as the gold standard in determining the accuracy of DSM diagnoses in adults and adolescents (Wittchen et al., 1997; Zanarini et al., 2000).

The German version of the Eating Disorder Inventory‐2 (EDI‐2) was used to assess psychopathological features of healthy adolescents and adolescents with eating disorders (Kappel et al., 2012). The EDI‐2 consists of 91 items on a 6‐point Likert scale and 11 subscales including drive for thinness, bulimia, body dissatisfaction, ineffectiveness, perfectionism, interpersonal distrust, interoceptive awareness, maturity fears, asceticism (provisional), impulse regulation (provisional) and social insecurity (provisional). The subscales add up to a total score indicate the severity of eating disorder pathology.

#### 2.2.2 Attachment classification

We used the AAP (George & West, 2012) to assess adolescent attachment status. This interview consists of a set of 8 picture stimuli (1 neutral and 7 attachment scenes) depicting important attachment‐related themes like illness, death, separation and solitude. Individuals were asked to tell a story about each picture by using a series of standardized questions: What happens in the scene? What are the characters thinking or feeling? What might happen next? Reliable AAP coders can classify four attachment representations according to the AAP manual (George & West, 2012):

1. secure attachment pattern (i.e., mutual enjoyment in attachment relationships and thoughtful self‐exploration)
2. insecure‐dismissing individuals (many deactivating elements, i.e., a lot of distance in relationships)
3. insecure‐preoccupied pattern (characterized by confusing material and negative emotions like anger, guilt and shame)
4. unresolved attachment (attachment dysregulation). Their narratives are characterized by an inability to protect themselves or seek protection or care from significant others when facing traumatic attachment‐related themes like abuse, death, emptiness, isolation or danger (Solomon & George, 2011).

In further analyses, we distinguished AN patients with unresolved from resolved attachment status (secure, insecure‐dismissing, insecure‐preoccupied).

### 2.3 MRI acquisition and processing

All participants underwent 3.0 Tesla MRI (Verio, Siemens, Erlangen, Germany) utilizing a predefined, standardized protocol. The parameters for coronal T1‐weighted 3D MPRAGE were as follows: repetition time (TR) 1950 ms; echo time (TE) 3.3 ms; flip angle 9°, in‐plane field of view 220 × 178 mm; slice thickness 1 mm; 160 contiguous transversal slices; voxel resolution .9 × .7 × 1 mm. Sequences were assessed by experienced neuroradiologists to exclude abnormal subclinical findings. All MRI data were visually inspected for artefacts arising from motion or instrument failure passed this quality control as well as the homogeneity control implemented in the CAT12 toolbox.

Whole brain analysis was conducted using an automated processing algorithm implemented in the Computational Anatomy Toolbox (CAT12; Structural Brain Mapping group, University of Jena, Germany) within SPM12 (Statistical Parametric Mapping, Institute of Neurology, London, UK) whilst running MATLAB 9.5 (R2018b; MathWorks, Natick, MA, USA). All high‐resolution T1‐weighted were bias‐field corrected, skull‐stripped, aligned to the Montreal Neurological Institute (MNI) standard space (MNI‐152 template) and segmented as grey, white matter and cerebrospinal fluid (Ashburner & Friston, 2005). Further, images were spatially normalized using the DARTEL algorithm (Ashburner, 2007). Spatially normalized segmented tissue maps were smoothed with a Gaussian kernel of 4 × 4 × 4 mm (full width at half maximum [FWHM]). A masking threshold of 10% was applied to reduce signal noise (Lenhart et al., 2020).

### 2.4 Statistical analysis

Statistical analyses were carried out using the statistical software package SPSS version 25 (SPSS Inc., Chicago, IL, USA). Normal distribution of our data was analysed with the Kolmogorov–Smirnov and Shapiro–Wilk test. Group differences between patient and control groups were assessed by Pearson's Chi‐square tests (marital status of parents, number of siblings, education, attachment status). For normal distributed variables like the eating disorder symptom severity, the independent‐samples *t* test was used to calculate group differences with significance levels set to *α* = .05. The Benjamini and Hochberg false discovery rate (FDR) was used for multiple comparisons correction (demographic and clinical characteristics) to control the false positive rate at 5%. Effect sizes were calculated using Cohen's (1988) conventions: small effect *d* = .2, medium effect *d* = .5 and large effect *d* = .8.

For voxel‐based analyses of the whole brain, a general linear model was set up to compare cross‐sectional and longitudinal data from the baseline with the follow‐up timepoint using a flexible factorial design implemented in SPM12 with total intracranial volume and age as nuisance variables. In the cross‐sectional analysis, the main effect of group (AN vs. HC) was used, and for within‐group analysis, the main effect of time was tested. In a separate analysis, the interaction of time × attachment group (individuals with an unresolved vs. a resolved attachment status) on GM volume. Additionally, BMI was included as a covariate in statistical models that investigated group differences on attachment status to reduce related variance. Inferences were made at *p* < .001 for group comparisons and *p* < .01 at cluster level for interaction analysis followed by correction for multiple comparisons via the family‐wise error (FWE) rate at *p* < .05 level.

## 3 RESULTS

### 3.1 Socio‐demographic and clinical values

Demographic and clinical characteristics are summarized in Tables 1 and 2. As expected, AN patients had higher scores on the EDI‐2 (*p* < .001) and a lower BMI (*p* < .001) compared with the HC group. Patients with resolved attachment status did not differ from those with an unresolved attachment status in terms of symptom severity as assessed with the EDI‐2 and the BMI. AN patients had a significantly lower BMI (*p* < .001) at baseline and follow‐up timepoint (*p* < .001) than HC. Patients and controls did not differ on socio‐demographic characteristics (number of siblings, marital status of their parents, occupation) and attachment classifications (outlined in Table 2).

**TABLE 1:** Demographic and clinical characteristics of the whole anorexia nervosa cohort, the unresolved and resolved attachment status groups and healthy controls at timepoint 1 (baseline)

	acAN cohort			HC cohort
	Whole cohort	Resolved attachment	Unresolved attachment	
Sample size	22	10	12	18
Age (years)	15.8 (1.2)*	16 (1.2)*	15.6 (1.3)*	17.7 (.7)*
BMI tp1 (kg/m^2^)	15.4 (1.4)*	15.5 (.9)*	15.5 (1.4)*	21.2 (1)*°
BMI tp2 (kg/m^2^)	17.8 (1)°	17.8 (1.2)°	17.7 (.7)°	‐
Duration of illness (months)	9.4 (6.8)	9.6 (4.8)	9.3 (8.3)	‐
EDI‐2 (total score)	298.6 (63)*	316 (44)*	293.4 (69)*	217.7 (58.4)*
Months between 1st and 2nd MRI scan	2.6 (.9)	2.9 (1)	2.4 (.8)	‐
TIV (mm^3^)	1336.5 (114.1)	1366.6 (122.3)	1309.9 (116.8)	1369.8 (93.9)

*Note*: Raw values are represented as mean (±1 standard deviation); the statistical tests are corrected for multiple comparisons (Holm–Sidak) in 5% significance level.

Abbreviations: acAN, acute anorexia nervosa patients at timepoint 1 (baseline); BMI, body mass index; EDI‐2, Eating Disorder Inventory 2; HC, healthy controls; MRI, magnetic resonance imaging; TIV, total intracranial volume; tp1, timepoint 1; tp2, timepoint 2.

* Significant differences between AN and HC cohorts (*p* < .001).

° Significant differences of BMI of AN patients at tp2 compared with HC (*p* < .001) (no significant differences were found between the attachment subgroups).

**TABLE 2:** Socio‐demographic characteristics and attachment distributions among the patient and the control group

	AN group	HC group	*χ* ^2^	Φ	*p*
	*n* = 22 (%)	*n* = 18 (%)			
Number of siblings					
Single child	4 (18.2)	2 (11.8)	4.296	.33	.231
One sibling	9 (40.9)	6 (35.3)			
Two siblings	3 (13.6)	7 (41.2)			
More than two siblings	6 (27.3)	2 (11.8)			
Marital status of parents					
Married/partnership	9 (40.9)	11 (61.1)	1.616	−2.01	.204
Single/divorced	13 (59.1)	7 (38.9)			
Occupation					
Attending school	21 (95.5)	17 (94.4)	.021	.02	.884
Employed/trainee	1 (4.5)	1 (5.6)			
Attachment classification					
Resolved/organized	10 (45.5)	14 (77.8)	4.310	−.33	.038
Unresolved/disorganized	12 (54.5)	4 (22.2)			

*Note*: Resolved, adolescents with a secure, an insecure‐dismissing or an insecure‐preoccupied attachment pattern; unresolved, adolescents with a disorganized attachment status that is related to attachment trauma; level of significance *p* ≤ .05.

Abbreviations: AN, anorexia nervosa; HC, healthy controls.

### 3.2 GM changes in female adolescents with AN before and after therapy

**FIGURE 1:**
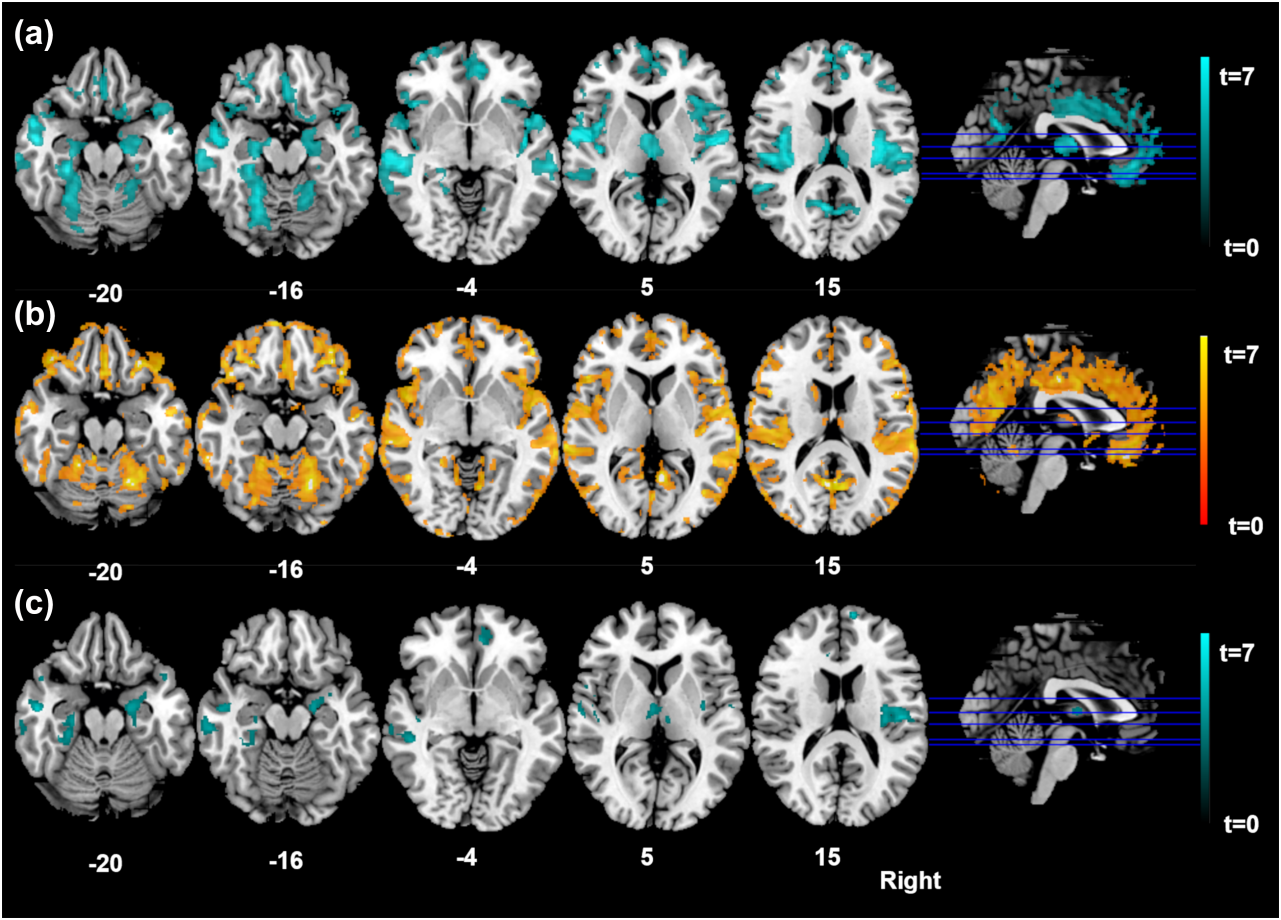
Statistical parametric mapping (*t*) intensity projection maps rendered onto a stereotactically normalized magnetic resonance imaging (MRI) scan, voxel cluster of the significant grey matter alterations in anorexia nervosa (statistical significance is thresholded at *p* < .001, family‐wise error [FWE] *p* < .05 corrected at the cluster level). (a) Grey matter decreases in anorexia nervosa patients relative to healthy controls at timepoint 1 (blue colour), (b) increases in anorexia nervosa patients from pre to post therapy (orange colour), (c) decreases in weight restored anorexia nervosa patients at timepoint 2 to relative to healthy controls (blue colour). The number at the bottom of each MRI scan corresponds to the *z* coordinate in Montreal Neurological Institute (MNI) space. The right side of the image corresponds to the right side of the brain

SPM localized extensive GM decreases throughout the brain in acute AN patients relative to HCs in several regions of extended association cortices including the insula and cingulate cortices (*p* < .001), inferior parietal lobule (*p* < .001 right‐hemispheric, *p* = .01 left‐hemispheric), frontal cortices including the orbitofrontal cortex (*p* < .001), fusiform gyrus (*p* < .001), precuneus (*p* < .001), cuneus (*p* < .001), parahippocampal gyrus (*p* < .001), cerebellar regions (*p* < .001) and the thalamus (*p* < .001) and the hippocampus and amygdala (*p* < .001 right‐hemispheric, *p* = .018 left‐hemispheric) (Table 3, Figure 1).

**TABLE 3:** Significant grey matter decreases in the 22 patients with acute anorexia nervosa compared with the 18 healthy control participants

	Cluster size (number of significant voxels)	MNI coordinates (centre of cluster)			*t* value	*p* value corrected at cluster level (FWE)	Height threshold
		*x*	*y*	*z*			
Left hemispheric							
Extended association cortices including the insula and cingulate cortices	10,650	−62 −54	−8 −8	−11 −17	9.8	<.001	.001
Inferior parietal lobule	242	−59	−36	48	5	.01	
Frontal cortices including the orbitofrontal cortex	455	−35 −27	38 35	−14 −12	6	<.001	
Fusiform gyrus and precuneus	5979	−21 −6	−65 −62	−15 17	6.8	<.001	
Cuneus	489	−30	−81	32	6	<.001	
Left thalamus extending to the right thalamus	1261	−3 8	−6 −20	8 17	6.3	<.001	
Parahippocampal gyrus spreading to the hippocampus and the amygdala	217	−24 −30	11 6	−26 −33	5.5	.018	
Cerebellar regions	2062	−33	−74	−48	7.2	<.001	
Right hemispheric							
Extended association cortices including the insula and cingulate cortices	15,558	38	−18	15	8.5	<.001	
	1338	60	−14	−9	6.1	<.001	
Inferior parietal lobule	531	56	−48	45	8	<.001	
Frontal cortices including the orbitofrontal cortex	1034	57 44	−6 −15	38 39	7.3	<.001	
Inferior frontal gyrus	581	56	11	26	7.3	<.001	
Inferior temporal gyrus	436	57	−14	−26	6.7	<.001	
Hippocampus spreading to the parahippocampal gyrus and the amygdala	801	26 20	−11 −2	−18 −15	5.1	<.001	
Fusiform gyrus	1268	20	−48	−14	5.8	<.001	
Cerebellar regions	2003	36	−45	−45	6.8	<.001	

Abbreviation: MNI, Montreal Neurological Institute.

Within‐group comparison of the 18 weight restored AN patients showed GM volume increases from Tp1 to Tp2 in most of these initially decreased regions. Significant GM increases were found in clusters of extended bilateral association cortices including the insula, cingulate cortices, orbitofrontal and cerebellar regions (*p* < .001), left inferior parietal lobule (*p* < .001), left pre‐ and postcentral gyrus (*p* < .001), right precentral gyrus (*p* < .013), anterior cingulate and right parahippocampal gyrus (*p* < .001), left cuneus (*p* < .004), bilateral temporal pole (*p* ≤ .001) and the thalamus bilaterally (*p* = .001). No significant GM increases were found in regions of the hippocampus and amygdala.

Overall, the extended character of GM volume reductions in AN patients was significantly less pronounced at Tp2. Regions that remained decreased in weight restored AN patients relative to HCs at Tp2 included the right medial frontal gyrus (*p* = .023), anterior and mid cingulate (*p* ≤ .002), frontotemporal cortices including the insula (*p* ≤ .009), right cerebellar regions (*p* < .001) and the right hippocampus and amygdala (*p* < .001) and the thalamus bilaterally (*p* ≤ .003) (Table 4, Figure 1). Several discrete brain regions that displayed significantly reduced GM volumes in acute AN were no longer significantly different from HC following weight restoration such as the orbitofrontal cortex, dorsolateral prefrontal cortex, the posterior cingulate cortex, precuneus, occipital and parietal association cortices on both sides, as well as the left hippocampus.

**TABLE 4:** Significant grey matter increases from timepoint 1 to timpoint 2 in the 18 anorexia nervosa patients and remaind grey matter decreases in the 18 weight restored anorexia nervosa patients compared to the 18 healthy control participants

	Cluster size (number of significant voxels)	MNI coordinates (centre of cluster)			*t* value	*p* value corrected at cluster level (FWE)	Height threshold
		*x*	*y*	*z*			
Significant grey matter increases in the 18 anorexia nervosa patients from pre to post therapy							
Left hemispheric							
Extended association cortices including the insula, cingulate cortices, orbitofrontal and cerebellar regions	35,954	−62 −9	−15 −65	24 −15	10.7	<.001	.001
Inferior parietal lobule	360	−33	−53	45	8.1	<.001	
Postcentral gyrus	445	−45	−29	53	7.7	<.001	
Precentral gyrus	115 194	−15 −35	−29 −24	66 53	7.3 6.3	.008	
Cuneus	131	−17	−102	−5	6.3	.004	
Temporal pole	240 116	−44 −41	5 21	−44 −41	6.1 5.7	<.001	
Frontal cortices	146	−24	2	53	6	.002	
Left thalamus extending to the right thalamus	150	−6 6	−12 −14	15 17	4.5	.001	
Right hemispheric							
Extended association cortices including the insula, cingulate cortices, orbitofrontal and cerebellar regions	35,954	63 50	−11 20	15 6	8.7	<.001	.001
Precentral gyrus	106	17	−27	68	6.2	.013	
Anterior cingulate and parahippocampal gyrus	356	0 15	9 −11	−9 −24	6.4	<.001	
Temporal pole	169	39	−14	−33	5.9	.001	
Significant grey matter decreases in the 18 weight restored anorexia nervosa patients at timepoint 2 compared to the 18 healthy control participants							
Left hemispheric							
Frontotemporal cortices including the insula	235 236 418	−53 −50 −62	−9 −3 −26	−21 8 −17	6.7 5.4 4.9	.009 .009 <.001	.001
Anterior and mid cingulate	308	−8	26	38	5	.002	
Fusiform and parahippocampal gyrus	393	−36	−32	−21	5.9	<.001	
Left thalamus extending to the right thalamus	285	−5 6	−9 −14	8 −2	4.7 4.6	.003	
Right hemispheric							
Frontotemporal cortices including the insula	873	44 35	−18 −18	20 17	6.3 5	.001	.001
Medial frontal gyrus	196	9	63	15	6.8	.023	
Anterior and mid cingulate	340	5	44	−6	5.1	.001	
Hippocampus spreading to the parahippocampal gyrus and the amygdala	419 265	21 59	−9 −12	−20 −30	5 5.9	<.001 .005	
Cerebellar regions	650	35	−45	−45	5.5	<.001	

Abbreviation: MNI, Montreal Neurological Institute.

### 3.3 Relationship between attachment status and GM recovery after therapy

**FIGURE 2:**
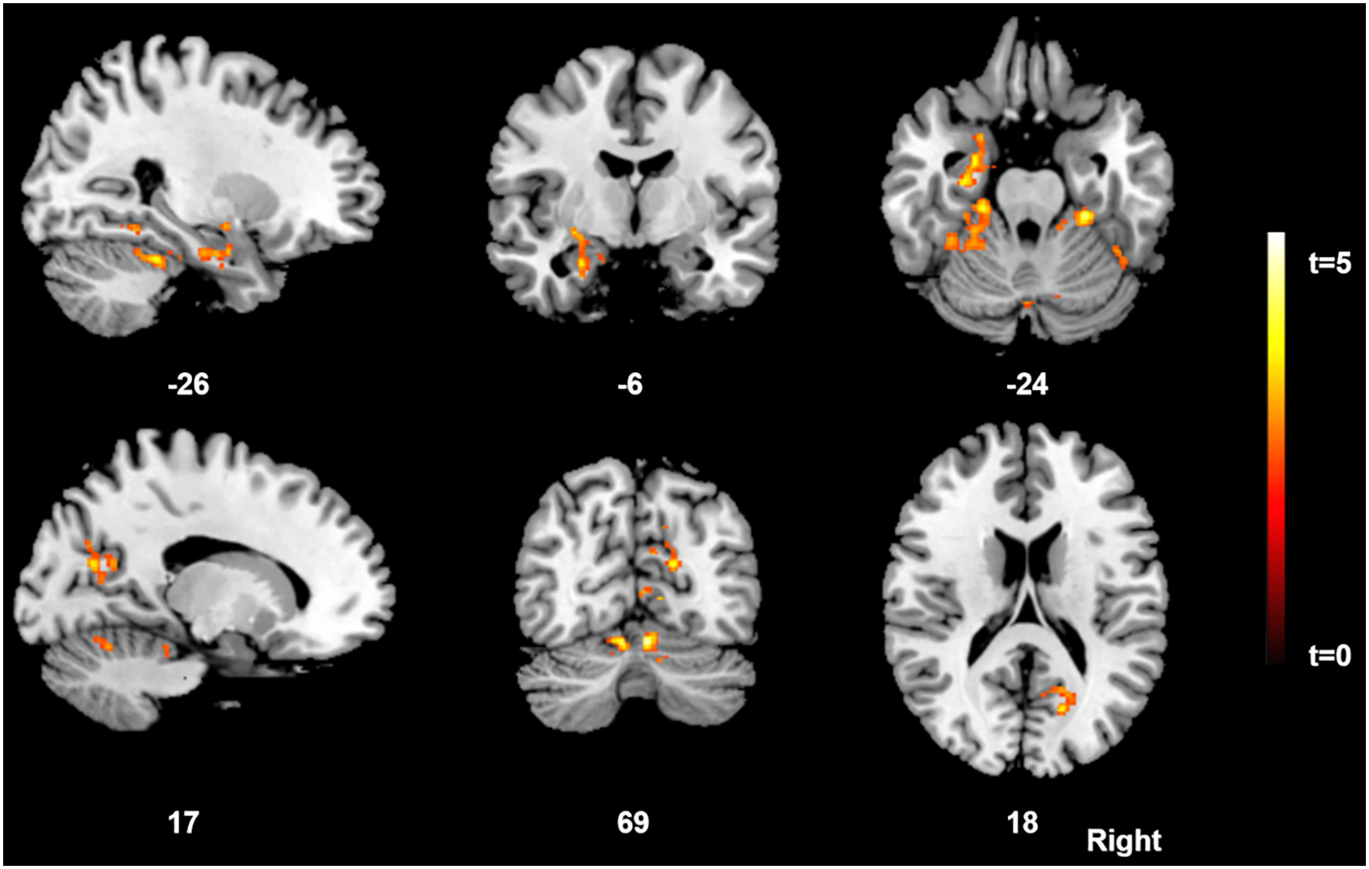
Statistical parametric mapping (*t*) intensity projection maps rendered onto a stereotactically normalized magnetic resonance imaging (MRI) scan, showing voxel clusters of the significant interaction of grey matter increases from the baseline to follow‐up timepoint in anorexia nervosa patients with a resolved/organized versus an unresolved/disorganized attachment pattern (statistical significance is thresholded at *p* < .01, family‐wise error [FWE] *p* < .05 corrected at the cluster level). The number at the bottom of each MRI scan corresponds to the *x*, *y* and *z* coordinate in Montreal Neurological Institute (MNI) space. The right side of the image corresponds to the right side of the brain

At Tp1, patients with a resolved and an unresolved attachment status did not differ on their GM volume. A significant interaction effect of time (Tp1 to Tp2) × attachment group (resolved vs. unresolved) on GM volume was evident in the left hippocampus and parahippocampal gyrus (*p* = .024), right fusiform gyrus (*p* = .013), right precuneus and cuneus spreading to cingulate cortices (*p* = .011) and cerebellar regions (*p* < .001) including the vermis (*p* = .013) (Table 5, Figure 2). The interaction effect was such that patients with the resolved attachment pattern showed more GM increases after therapy relative to the unresolved group. No significant GM increases were found for the unresolved compared with the resolved group at Tp1 and Tp2.

**TABLE 5:** Interaction of grey matter volumes showing relative increases from pre to post therapy in the 8 anorexia nervosa patients without attachment trauma versus the 10 patients with attachment trauma

	Cluster size (number of significant voxels)	MNI coordinates (centre of cluster)			*t* value	*p* value corrected at cluster level (FWE)	Height threshold
		*x*	*y*	*z*			
Left hemispheric							
Hippocampus and parahippocampal gyrus	348	−35 −26	−24 −8	−18 −24	4.86	.024	.01
Vermis	382	−3	−54	−15	4.8	.013	
Cerebellar regions	646	−20	−30	−21	5.43	<.001	
Right hemispheric							
Right fusiform gyrus	382	23	−36	−21	3.64	.013	
Right precuneus and cuneus spreading to the posterior cingulate	391	17 3	−69 −68	18 6	4.29	.011	
Cerebellar regions	548	5 23	−69 −68	−17 −20	5.18	<.001	

Abbreviation: MNI, Montreal Neurological Institute.

## 4 DISCUSSION

Despite the growing number of neuroimaging studies that investigated structural brain alterations in acute AN, there is still a relative lack of longitudinal studies focusing on adolescent age groups from 14 to 18 years. The present study found that acutely ill patients with AN revealed GM volume decreases in extended association cortices including the insula and cingulate structures, inferior parietal lobule, frontal cortices including the orbitofrontal gyrus, fusiform gyrus, precuneus/cuneus and parahippocampal/fusiform gyrus, cerebellar areas, and subcortical regions such as the thalamus, hippocampus and amygdala. After short‐term weight restoration and inpatient psychiatric treatment, voxel‐based morphometry yielded volume restoration in several of these regions with a relative sparing in some areas including hippocampal and parahippocampal cortices as well as the amygdala. In this first study to investigate influences of attachment status on region‐specific brain recovery in adolescents with AN, interaction analysis revealed that patients with an unresolved attachment pattern showed less GM volume increases in regions of the right precuneus/posterior cingulate, as well as the left hippocampus, left parahippocampal gyrus and cerebellar areas after weight recovery compared with patients with a resolved attachment status.

The overall pattern of GM reductions is consistent with previous investigations that applied voxel‐based morphometry in adolescent patients with acute AN (Bernardoni et al., 2016; Bomba et al., 2015; Castro‐Fornieles et al., 2010; Mainz et al., 2012). After inpatient treatment, longitudinal analysis yielded nearly complete GM volume recovery in most of these cortical regions, particularly including the orbitofrontal cortex, prefrontal and insular cortices and the precuneus, whereas a number of regions such the right medial frontal gyrus, anterior and mid cingulate, right hippocampus, amygdala and caudate nuclei did not display any significant GM increases and remained decreased after weight recovery. The majority of our findings are in line with one of the few existing studies that followed up a subset of 10 adolescent AN patients after weight restoration (Martin Monzon et al., 2017). In contrast to this previous study, the extent of the GM decreases at Tp2 in our study was more extended as several additional regions such as the the mid cingulate, right cerebellar and insular cortices as well as parts of the thalamus bilaterally remained decreased. Previous analyses of acutely ill AN patients reported reduced GM volumes in the anterior cingulate to persist after weight restoration therapy (Friederich et al., 2012) and that the anterior cingulated is related to symptom severity of AN (Mühlau et al., 2007). Regarding structural alterations of the caudate nuclei in AN, there is a certain inconsistency in literature, as both GM volume increases (Mainz et al., 2012) and decreases (Martin Monzon et al., 2017) after weight restoration therapy have been shown. Inconsistencies may at least partially rely on varying participant's characteristics such as the history and duration of eating disorders in different study cohorts. The more widespread pattern of continuous GM decreases at Tp2 may be constituted by several reasons. First, the recovery period of approximately 2 months of inpatient treatment might be relatively short compared with other studies. Our specialized inpatient treatment of AN patients provides medical and psychiatric stabilization for adolescents who are in the most acute phase of their disease course. After reaching a BMI‐for‐age ≥ 5th percentile, patients received outpatient treatment to provide a less restrictive setting that allowed them to remain engaged in school/work, stay connected to their family members and social supports (i.e., peer groups) whilst recovering from AN. Even though our adolescent cohort showed a BMI in the lower normal range after hospitalization, their BMI might have been lower at the Tp2 compared with the BMI of patients reported in other studies (Martin Monzon et al., 2017). Second, several brain regions might need more time to recover, and thus, it would be interesting for future research to conduct additional functional and quantitative MRI measurements after outpatient treatment and longer follow‐up periods. This might provide new insights into whether certain brain regions remain irreversibly altered after complete recovery from AN. Third, decreases in brain regions might be a result of a patient's underlying psychopathological profile.

In the second part of this study, AN patient groups were contrasted to each other regarding their attachment status—a novel analysis on the literature. The AN patient group with a preexisting unresolved attachment status revealed less GM volume recovery after therapy in several brain regions that are critically involved in emotion regulation compared with those individuals with a resolved attachment pattern. One of these regions was the precuneus and adjacent cingulate cortices, which is a highly interconnected region receiving inputs from the anterior and mid cingulate as well as from thalamic nerve fibres (Herrero et al., 2002). As a major hub of the default mode network (Hagmann et al., 2008), the posterior cingulate together with the precuneus combines body‐orientation inputs from the anterior cingulate with contextual information and sends projections to the posterior part of the inferior parietal lobule, the dorsolateral prefrontal and the orbitofrontal cortex to create a final representation of one's own body in spatial context (Vogeley & Fink, 2003). Structural abnormalities in these regions may alter the way of reflecting body dimensions in adolescents with AN—meaning that lower GM volume in these regions might be associated with greater dissatisfaction with body weight (Cavanna & Trimble, 2006). Decreased volumes in this brain region were further associated with higher distractibility and less attention to inner mental processes involving self‐evaluation, reflection upon one's mental state, episodic memory retrieval, referential processing and first‐person perspective (Cavanna & Trimble, 2006; Vogt et al., 2006). In line with our observation, other studies also reported smaller GM volumes in the precuneus in individuals with severe insecure attachment styles (Jin et al., 2016) and greater experiences of affective losses (Acosta et al., 2018; Benetti et al., 2010). Individuals with an unresolved attachment pattern are unable to find an alternative source of security that regulates their emotions in the view of attachment‐related stressors. Consequently, they remain in a state of arousal, threat and helplessness (Buchheim & George, 2011) resulting in different emotional, behavioural and social difficulties like feeling detached from other people and being unable to regulate anger, anxiety and stress (Rahim, 2014). These impairments in AN patients might be linked to the less GM recovery in the precuneus after weight restoration.

A second region with lower GM volume increases after inpatient treatment in AN patients with unresolved attachment trauma covered extended parts of the cerebellum. Structural and functional cerebellar abnormalities have been reported in several psychiatric disorders that are associated with severe emotional dysregulation such as bipolar, unipolar affective and anxiety disorders (Baldaçara et al., 2008). Furthermore, one study suggested that the impact of traumatic experiences like major losses, which typically lead to emotion dysregulation in some patients, on GM volume in the cerebellum is mediated by individual differences in attachment style (Benetti et al., 2010). Thus, poor emotion regulation capacities in response to interpersonal stressors observed in adolescents with an unresolved attachment status might be linked to abnormalities in the cerebellum after inpatient treatment in the present cohort of AN patients.

Third, we found smaller GM volume recovery in the left hippocampus and parahippocampal gyrus in unresolved compared with resolved AN patients. The hippocampus is considered as one of the most stress‐sensitive structures in the human brain (Bernard et al., 2014) and involved in the hypothalamic–pituitary–adrenal axis responsiveness to stress. Several studies showed smaller hippocampal volumes in patients with traumatic attachment histories (Wignall et al., 2004) and experiences of maltreatment (Riem et al., 2015). Thus, patients with AN and unresolved attachment representations might be more vulnerable to negative emotional stimuli regarding their mental representations of major losses or other traumatic emotional experiences. Less GM volume increases in the hippocampus may represent a less effective hypothalamic–pituitary–adrenal axis response resulting in a lower threshold to experience stress through memories of attachment trauma (van Hoof et al., 2019). Clinicians often encounter patients with AN and with a lack of confidence to deal with negative emotions (Burns et al., 2012). Particularly, patients with an unresolved attachment status tend to deny severe ruptures in the parent–child attachment bond and associated feelings to avoid abandonment or disruptions in their attachment relationships. They do not openly communicate their feelings, interests and needs (Latzer & Hochdorf, 2005). Their eating behaviour and resentment of food may be a maladaptive way to regain control over their lives and replaces their secure base needs (Latzer & Hochdorf, 2005). These patients can be particularly challenging in the clinical setting as they more often drop out of therapy and demonstrate an adverse outcome (Bizzi et al., 2015).

In sum, our results suggest that severe interpersonal impairments also become apparent in therapy‐related GM plasticity of brain structures that are involved in emotion regulation, processing of inner mental processes, fear of gaining weight and body dissatisfaction. In other words, less region‐specific GM recovery in patients with an unresolved attachment status in AN might be an indicator of persistent emotion dysregulation after traditional psychotherapeutic and nutritional treatment. Therefore, AN patients might additionally benefit from attachment‐based treatments (Kobak & Kerig, 2015), which focus on traumatic attachment‐related material. Such treatments might have the potential to foster efficacious change mechanisms that help AN patients to gain attachment security in relying on others and enjoying reciprocal and collaborative close relationships from which to explore the world (Levy et al., 2018).

The present study has some limitations. Our cohort was focused on adolescent patients ≤ 18 years, and thus, the presently found changes may not be found in cohorts with older patients or longer disease durations. Further, our sample consisted of hospitalized female patients with severe AN symptomatology. Studies that include outpatients who have mild or moderate levels of AN might lead to different results. Future studies investigating differences between inpatient and outpatient cohorts as well as differences between eating disorder subgroups are needed to draw further conclusions on brain alterations before and after therapy. Although attachment patterns are unlikely to shift from insecure to secure during the course of short‐term treatment, exploring whether changes of attachment status during the treatment occur may represent an exciting field for future research. The distribution of resolved and unresolved attachment patterns is analogous to the distribution reported in other meta‐analyses of adolescent attachment in community samples (for a review, see Gander et al., 2015; Bakermans‐Kranenburg, & van IJzendoorn, M. H., 2009).

## 5 CONCLUSION

In sum, our findings of cortical decreases in adolescents with acute AN and the partial recovery after combined psychotherapeutic and nutritional therapy contribute to the understanding of the heterogeneous AN literature. Initial GM reductions in regions that are considered as important regions for AN symptomatology were shown to partially recover. Nevertheless, several brain regions remained at decreased levels after short‐term therapy. In this context, our results suggest that attachment status might be an underlying protective factor for better GM recovery in certain brain regions that were associated with executive and cognitive functions as well as the regulation of affective states. Therefore, these structural differences may be related to psychopathology and outcome. Specific treatments focusing on attachment related issues and trauma might be particularly helpful to improve the psychological and the biological outcome in patients with AN and should be addressed in future studies.

## CONFLICT OF INTEREST

The authors declare that they have no known competing financial interests or personal relationships that could have appeared to influence the work reported in this paper.

### PEER REVIEW

The peer review history for this article is available at https://publons.com/publon/10.1111/ejn.15614.

## Data Availability

The datasets used and analysed for the present paper can be made available on request to the corresponding author due to privacy/ethical restrictions.

## References

[ejn15614-bib-0001] Acosta, H. , Jansen, A. , Nuscheler, B. , & Kircher, T. (2018). A voxel‐based morphometry study on adult attachment style and affective loss. Neuroscience, 392, 219–229. 10.1016/j.neuroscience.2018.06.045 30005995

[ejn15614-bib-0003] Ashburner, J. (2007). A fast diffeomorphic image registration algorithm. NeuroImage, 38(1), 95–113. 10.1016/j.neuroimage.2007.07.007 17761438

[ejn15614-bib-0004] Ashburner, J. , & Friston, K. J. (2005). Unified segmentation. NeuroImage, 26(3), 839–851. 10.1016/j.neuroimage.2005.02.018 15955494

[ejn15614-bib-0005] Bakermans‐Kranenburg, M. J. , & van IJzendoorn, M. H. (2009). The first 10,000 Adult Attachment Interviews: distributions of adult attachment representations in clinical and non‐clinical groups. Attachment & Human Development, 11(3), 223–263. 10.1080/14616730902814762 19455453

[ejn15614-bib-0006] Baldaçara, L. , Borgio, J. G. F. , de Lacerda, A. L. T. , & Jackowski, A. P. (2008). Cerebellum and psychiatric disorders. Revista Brasileira de Psiquiatria, 30(3), 281–289. 10.1590/S1516-44462008000300016 18833430

[ejn15614-bib-0007] Benetti, S. , McCrory, E. , Arulanantham, S. , de Sanctis, T. , McGuire, P. , & Mechelli, A. (2010). Attachment style, affective loss and gray matter volume: A voxel‐based morphometry study. Human Brain Mapping, 31(10), 1482–1489. 10.1002/hbm.20954 20127871 PMC6870872

[ejn15614-bib-0008] Bernard, K. , Lind, T. , & Dozier, M. (2014). Neurobiological consequences of neglect and abuse. In J. E. Korbin & K. D. Krugman (Eds.), Handbook of Child Maltreatment: Contemporary Issues in Research and Policy (pp. 205–223). Springer. 10.1007/978-94-007-7208-3_11

[ejn15614-bib-0009] Bernardoni, F. , King, J. A. , Geisler, D. , Stein, E. , Jaite, C. , Nätsch, D. , Tam, F. I. , Boehm, I. , Seidel, M. , Roessner, V. , & Ehrlich, S. (2016). Weight restoration therapy rapidly reverses cortical thinning in anorexia nervosa: A longitudinal study. NeuroImage, 130, 214–222. 10.1016/j.neuroimage.2016.02.003 26876474

[ejn15614-bib-0010] Bizzi, F. , Cavanna, D. , Castellano, R. , & Pace, C. S. (2015). Children's mental representations with respect to caregivers and post‐traumatic symptomatology in somatic symptom disorders and disruptive behavior disorders. Frontiers in Psychology, 6, 1125. 10.3389/fpsyg.2015.01125 26284022 PMC4522510

[ejn15614-bib-0011] Bomba, M. , Riva, A. , Morzenti, S. , Grimaldi, M. , Neri, F. , & Nacinovich, R. (2015). Global and regional brain volumes normalization in weight‐recovered adolescents with anorexia nervosa: Preliminary findings of a longitudinal voxel‐based morphometry study. Neuropsychiatric Disease and Treatment, 11, 637–645. 10.2147/NDT.S73239 25834442 PMC4358418

[ejn15614-bib-0012] Buchheim, A. , & Diamond, D. (2018). Attachment and borderline personality disorder. The Psychiatric Clinics of North America, 41(4), 651–668. 10.1016/j.psc.2018.07.010 30447730

[ejn15614-bib-0013] Buchheim, A. , & George, C. (2011). Attachment disorganization in borderline personality disorder and anxiety disorder. In J. Solomon & C. George (Eds.), Disorganized Attachment and Caregiving (pp. 343–382). The Guilford Press.

[ejn15614-bib-0015] Burns, E. E. , Fischer, S. , Jackson, J. L. , & Harding, H. G. (2012). Deficits in emotion regulation mediate the relationship between childhood abuse and later eating disorder symptoms. Child Abuse & Neglect: The International Journal, 36(1), 32–39. 10.1016/j.chiabu.2011.08.005 22265934

[ejn15614-bib-0016] Castro‐Fornieles, J. , Caldú, X. , Andrés‐Perpiñá, S. , Lázaro, L. , Bargalló, N. , Falcón, C. , Plana, M. T. , & Junqué, C. (2010). A cross‐sectional and follow‐up functional MRI study with a working memory task in adolescent anorexia nervosa. Neuropsychologia, 48(14), 4111–4116. 10.1016/j.neuropsychologia.2010.10.003 20933530

[ejn15614-bib-0017] Cavanna, A. E. , & Trimble, M. R. (2006). The precuneus: A review of its functional anatomy and behavioural correlates. Brain: A Journal of Neurology, 129(3), 564–583. 10.1093/brain/awl004 16399806

[ejn15614-bib-0018] de Paoli, T. , Fuller‐Tyszkiewicz, M. , Halliwell, E. , Puccio, F. , & Krug, I. (2017). Social rank and rejection sensitivity as mediators of the relationship between insecure attachment and disordered eating. European Eating Disorders Review, 25(6), 469–478. 10.1002/erv.2537 28752904

[ejn15614-bib-0019] Friederich, H.‐C. , Walther, S. , Bendszus, M. , Biller, A. , Thomann, P. , Zeigermann, S. , Katus, T. , Brunner, R. , Zastrow, A. , & Herzog, W. (2012). Grey matter abnormalities within cortico‐limbic‐striatal circuits in acute and weight‐restored anorexia nervosa patients. NeuroImage, 59(2), 1106–1113. 10.1016/j.neuroimage.2011.09.042 21967727

[ejn15614-bib-0020] Gander, M. , Diamond, D. , Buchheim, A. , & Sevecke, K. (2018). Use of the adult attachment projective picture system in the formulation of a case of an adolescent refugee with PTSD. Journal of Trauma & Dissociation, 19(5), 572–595. 10.1080/15299732.2018.1451803 29547072

[ejn15614-bib-0022] Gander, M. , Sevecke, K. , & Buchheim, A. (2015). Eating disorders in adolescence: Attachment issues from a developmental perspective. Frontiers in Psychology, 6, 1136. 10.3389/fpsyg.2015.01136 26321974 PMC4530258

[ejn15614-bib-0023] Gander, M. , Sevecke, K. , & Buchheim, A. (2018). Disorder‐specific attachment characteristics and experiences of childhood abuse and neglect in adolescents with anorexia nervosa and a major depressive episode. Clinical Psychology & Psychotherapy, 25(6), 894–906. 10.1002/cpp.2324 30216616 PMC6585713

[ejn15614-bib-0024] George, C. , & West, M. (2001). The development and preliminary validation of a new measure of adult attachment: The adult attachment projective. Attachment & Human Development, 3(1), 30–61. 10.1080/14616730010024771 11708383

[ejn15614-bib-0026] George, C. , & West, M. L. (2012). The Adult Attachment Projective Picture System: Attachment theory and assessment in adults. Guilford Press. 10.1080/00223891.2011.594133 21859280

[ejn15614-bib-0027] Hagmann, P. , Cammoun, L. , Gigandet, X. , Meuli, R. , Honey, C. J. , Wedeen, V. J. , & Sporns, O. (2008). Mapping the structural core of human cerebral cortex. PLoS Biology, 6(7), e159. 10.1371/journal.pbio.0060159 18597554 PMC2443193

[ejn15614-bib-0028] Herpertz‐Dahlmann, B. (2015). Adolescent eating disorders: Update on definitions, symptomatology, epidemiology, and comorbidity. Child and Adolescent Psychiatric Clinics of North America, 24(1), 177–196. 10.1016/j.chc.2014.08.003 25455581

[ejn15614-bib-0029] Herrero, M.‐T. , Barcia, C. , & Navarro, J. M. (2002). Functional anatomy of thalamus and basal ganglia. Child's Nervous System: ChNS: Official Journal of the International Society for Pediatric Neurosurgery, 18(8), 386–404. 10.1007/s00381-002-0604-1 12192499

[ejn15614-bib-0030] Hudson, J. I. , Hiripi, E. , Pope, H. G. Jr. , & Kessler, R. C. (2007). The prevalence and correlates of eating disorders in the National Comorbidity Survey Replication. Biological Psychiatry, 61(3), 348–358. 10.1016/j.biopsych.2006.03.040 16815322 PMC1892232

[ejn15614-bib-0031] Jin, X. , Zhong, M. , Yao, S. , Cao, X. , Tan, C. , Gan, J. , Zhu, X. , & Yi, J. (2016). A voxel‐based morphometric MRI study in young adults with borderline personality disorder. PLoS ONE, 11(1), e0147938. 10.1371/journal.pone.0147938 26808504 PMC4726531

[ejn15614-bib-0032] Joos, A. , Hartmann, A. , Glauche, V. , Perlov, E. , Unterbrink, T. , Saum, B. , Tüscher, O. , Tebartz van Elst, L. , & Zeeck, A. (2011). Grey matter deficit in long‐term recovered anorexia nervosa patients. European Eating Disorders Review, 19(1), 59–63. 10.1002/erv.1060 21038322

[ejn15614-bib-0033] Kappel, V. , Thiel, A. , Holzhausen, M. , Jaite, C. , Schneider, N. , Pfeiffer, E. , Lehmkuhl, U. , & Salbach‐Andrae, H. (2012). Eating disorder Inventory‐2 (EDI‐2) Normierung an einer stichprobe normalgewichtiger schüler im alter von 10 bis 20 jahren und an patientinnen mit anorexia nervosa = eating disorder inventory (EDI‐2): Normative data among 10 to 20 year old German girls and boys. Diagnostica, 58(3), 127–144. 10.1026/0012-1924/a000069

[ejn15614-bib-0034] Kaye, W. H. , Fudge, J. L. , & Paulus, M. (2009). New insights into symptoms and neurocircuit function of anorexia nervosa. Nature Reviews Neuroscience, 10(8), 573–584. 10.1038/nrn2682 19603056 PMC13038070

[ejn15614-bib-0035] King, J. A. , Frank, G. K. W. , Thompson, P. M. , & Ehrlich, S. (2018). Structural neuroimaging of anorexia nervosa: Future directions in the quest for mechanisms underlying dynamic alterations. Biological Psychiatry, 83(3), 224–234. 10.1016/j.biopsych.2017.08.011 28967386 PMC6053269

[ejn15614-bib-0036] King, J. A. , Geisler, D. , Ritschel, F. , Boehm, I. , Seidel, M. , Roschinski, B. , Soltwedel, L. , Zwipp, J. , Pfuhl, G. , Marxen, M. , Roessner, V. , & Ehrlich, S. (2015). Global cortical thinning in acute anorexia nervosa normalizes following long‐term weight restoration. Biological Psychiatry, 77(7), 624–632. 10.1016/j.biopsych.2014.09.005 25433902

[ejn15614-bib-0037] Kobak, R. R. , & Kerig, P. K. (2015). Introduction to the special issue: Attachment‐based treatments for adolescents. Attachment & Human Development, 17(2), 111–118. 10.1080/14616734.2015.1006382 25833287

[ejn15614-bib-0038] Laible, D. (2007). Attachment with parents and peers in late adolescence: Links with emotional competence and social behavior. Personality and Individual Differences, 43(5), 1185–1197. 10.1016/j.paid.2007.03.010

[ejn15614-bib-0039] Latzer, Y. , & Hochdorf, Z. (2005). Dying to be thin: Attachment to death in anorexia nervosa. TheScientificWorldJOURNAL, 5, 820–827. 10.1100/tsw.2005.95 16200328 PMC5936518

[ejn15614-bib-0040] Lenhart, L. , Steiger, R. , Waibel, M. , Mangesius, S. , Grams, A. E. , Singewald, N. , & Gizewski, E. R. (2020). Cortical reorganization processes in meditation naïve participants induced by 7 weeks focused attention meditation training. Behavioural Brain Research, 395, 112828. 10.1016/j.bbr.2020.112828 32745662

[ejn15614-bib-0041] Levy, K. N. (2013). Introduction: Attachment theory and psychotherapy. Journal of Clinical Psychology, 69(11), 1133–1135. 10.1002/jclp.2240 24122423

[ejn15614-bib-0042] Levy, K. N. , Kivity, Y. , Johnson, B. N. , & Gooch, C. V. (2018). Adult attachment as a predictor and moderator of psychotherapy outcome: A meta‐analysis. Journal of Clinical Psychology, 74, 1996–2013. 10.1002/jclp.22685 30238450

[ejn15614-bib-0043] Mainz, V. , Schulte‐Rüther, M. , Fink, G. R. , Herpertz‐Dahlmann, B. , & Konrad, K. (2012). Structural brain abnormalities in adolescent anorexia nervosa before and after weight recovery and associated hormonal changes. Psychosomatic Medicine, 74(6), 574–582. 10.1097/PSY.0b013e31824ef10e 22511729

[ejn15614-bib-0044] Martin Monzon, B. , Henderson, L. A. , Madden, S. , Macefield, V. G. , Touyz, S. , Kohn, M. R. , Clarke, S. , Foroughi, N. , & Hay, P. (2017). Grey matter volume in adolescents with anorexia nervosa and associated eating disorder symptoms. European Journal of Neuroscience, 46(7), 2297–2307. 10.1111/ejn.13659 28833732

[ejn15614-bib-0045] Maxwell, C. , McGeer, A. , Tai, K. F. Y. , & Sermer, M. (2017). No. 225‐management guidelines for obstetric patients and neonates born to mothers with suspected or probable severe acute respiratory syndrome (SARS). Journal of Obstetrics and Gynaecology Canada: JOGC = Journal d'obstetrique et Gynecologie du Canada: JOGC, 39(8), e130–e137. 10.1016/j.jogc.2017.04.024 PMC7105038 28729104

[ejn15614-bib-0046] Mühlau, M. , Gaser, C. , Ilg, R. , Conrad, B. , Leibl, C. , Cebulla, M. H. , Backmund, H. , Gerlinghoff, M. , Lommer, P. , Schnebel, A. , Wohlschläger, A. M. , Zimmer, C. , & Nunnemann, S. (2007). Gray matter decrease of the anterior cingulate cortex in anorexia nervosa. The American Journal of Psychiatry, 164(12), 1850–1857. 10.1176/appi.ajp.2007.06111861 18056240

[ejn15614-bib-0047] Petermann, F. , & Petermann, U. (2008). Hamburg‐Wechsler‐Intelligenztest für Kinder IV (HAWIK‐IV). Huber.

[ejn15614-bib-0048] Phillips, M. L. , Drevets, W. C. , Rauch, S. L. , & Lane, R. (2003a). Neurobiology of emotion perception I: The neural basis of normal emotion perception. Biological Psychiatry, 54, 504–514. 10.1016/S0006-3223(03)00168-9 12946879

[ejn15614-bib-0049] Phillips, M. L. , Drevets, W. C. , Rauch, S. L. , & Lane, R. (2003b). Neurobiology of emotion perception II: Implications for major psychiatric disorders. Biological Psychiatry, 54, 515–528. 10.1016/S0006-3223(03)00171-9 12946880

[ejn15614-bib-0050] Rahim, M. (2014). Developmental trauma disorder: An attachment‐based perspective. Clinical Child Psychology and Psychiatry, 19(4), 548–560. 10.1177/1359104514534947 24835949

[ejn15614-bib-0051] Riem, M. M. E. , Alink, L. R. A. , Out, D. , van Ijzendoorn, M. H. , & Bakermans‐Kranenburg, M. J. (2015). Beating the brain about abuse: Empirical and meta‐analytic studies of the association between maltreatment and hippocampal volume across childhood and adolescence. Development and Psychopathology, 27(2), 507–520. 10.1017/S0954579415000127 25997768

[ejn15614-bib-0052] Ringer, F. , & Crittenden, P. M. (2007). Eating disorders and attachment: The effects of hidden family processes on eating disorders. European Eating Disorders Review, 15(2), 119–130. 10.1002/erv.761 17676680

[ejn15614-bib-0053] Seitz, J. , Bühren, K. , von Polier, G. G. , Heussen, N. , Herpertz‐Dahlmann, B. , & Konrad, K. (2014). Morphological changes in the brain of acutely ill and weight‐recovered patients with anorexia nervosa. A meta‐analysis and qualitative review [Hirnmorphologische Veränderungen in akut kranken und gewichtsrehabilitierten Patientinnen mit anorexia nervosa ‐ Meta‐analyse und qualitativer review]. Zeitschrift für Kinder‐ und Jugendpsychiatrie und Psychotherapie, 42(1), 7–18. 10.1024/1422-4917/a000265 24365959

[ejn15614-bib-0054] Seitz, J. , Herpertz‐Dahlmann, B. , & Konrad, K. (2016). Brain morphological changes in adolescent and adult patients with anorexia nervosa. Journal of Neural Transmission, 123(8), 949–959. 10.1007/s00702-016-1567-9 27188331

[ejn15614-bib-0055] Smink, F. R. E. , van Hoeken, D. , & Hoek, H. W. (2012). Epidemiology of eating disorders: Incidence, prevalence and mortality rates. Current Psychiatry Reports, 14(4), 406–414. 10.1007/s11920-012-0282-y 22644309 PMC3409365

[ejn15614-bib-0056] Solomon, J. , & George, C. (2011). The disorganized attachment‐caregiving system: Dysregulation of adaptive processes at multiple levels. In J. Solomon & C. George (Eds.), Disorganized Attachment and Caregiving (pp. 3–24). The Guilford Press.

[ejn15614-bib-0057] Swanson, S. A. , Crow, S. J. , le Grange, D. , Swendsen, J. , & Merikangas, K. R. (2011). Prevalence and correlates of eating disorders in adolescents. Results from the national comorbidity survey replication adolescent supplement. Archives of General Psychiatry, 68(7), 714–723. 10.1001/archgenpsychiatry.2011.22 21383252 PMC5546800

[ejn15614-bib-0058] Titova, O. E. , Hjorth, O. C. , Schiöth, H. B. , & Brooks, S. J. (2013). Anorexia nervosa is linked to reduced brain structure in reward and somatosensory regions: A meta‐analysis of VBM studies. BMC Psychiatry, 13, 110. 10.1186/1471-244X-13-110 23570420 PMC3664070

[ejn15614-bib-0059] van Hoof, M. J. , Riem, M. , Garrett, A. , Pannekoek, N. , van der Wee, N. , van IJdenzoorn, M. , & Vermeiren, R. (2019). Unresolved‐disorganized attachment is associated with smaller Hippocampus and increased functional connectivity beyond psychopathology. Journal of Traumatic Stress, 32(5), 742–752. 10.1002/jts.22432 31361352 PMC6851754

[ejn15614-bib-0060] Vogeley, K. , & Fink, G. R. (2003). Neural correlates of the first‐person‐perspective. Trends in Cognitive Sciences, 7(1), 38–42. 10.1016/S1364-6613(02)00003-7 12517357

[ejn15614-bib-0061] Vogt, B. A. , Vogt, L. , & Laureys, S. (2006). Cytology and functionally correlated circuits of human posterior cingulate areas. NeuroImage, 29(2), 452–466. 10.1016/j.neuroimage.2005.07.048 16140550 PMC2649771

[ejn15614-bib-0062] Wignall, E. L. , Dickson, J. M. , Vaughan, P. , Farrow, T. F. D. , Wilkinson, L. D. , Hunter, M. D. , & Woodruff, P. W. R. (2004). Smaller hippocampal volume in patients with recent‐onset posttraumatic stress disorder. Biological Psychiatry, 56(11), 832–836. 10.1016/j.biopsych.2004.09.015 15576059

[ejn15614-bib-0063] Wittchen, H.‐U. , Zaudig, M. , & Fydrich, T. (1997). Strukturiertes klinisches Interview für DSM‐IV (SKID). Hogrefe.

[ejn15614-bib-0064] Zanarini, M. C. , Skodol, A. E. , Bender, D. , Dolan, R. , Sanislow, C. , Schaefer, E. , Morey, L. C. , Grilo, C. M. , Shea, M. T. , McGlashan, T. H. , & Gunderson, J. G. (2000). The collaborative longitudinal personality disorders study: Reliability of axis I and II diagnoses. Journal of Personality Disorders, 14(4), 291–299. 10.1521/pedi.2000.14.4.291 11213787

